# On Hallucinations in Insanity

**Published:** 1862-04

**Authors:** A. Brierre de Boismont


### Art. V.—ON HALLUCINATIONS IN INSANITY.

By A. Brierre de Boismont.*

Although hallucination may exist alone and independently of any complication, it is not less true that it is much more frequently observed in connexion with one or other of nearly all the generally admitted forms of mental alienation. According to Esquirol, amongst a hundred lunatics, eighty at least are subject to hallucination. More recent researches, however, give a lower average. It is amongst monomaniacs especially that hallucination is manifested, which may be due either to the form of the disease, or to the fact that its discovery is easier in such cases. The latter reason does not always apply; for there are melancholic patients who preserve an obstinate silence for years, and with respect to whom chance alone reveals the secret. It may be laid down, however, as a general rule, that the more singular and eccentric the actions of lunatics appear, the greater reason there is to consider them as the result of hallucinations or illusions.

"I saw," states Marc, "in the Asylum of Dr. Pressat, a middle-aged man whom a reverse of fortune had rendered melancholic. For several years he had not spoken a word, and his sole occupation consisted in smelling and licking the walls of his room and the threshold of the door, sometimes for hours together. No one was able to explain the motives of actions so extravagant and at the same time so painful to witness, and of which the frequency and duration had produced numerous deep impressions in the plaster walls of the chamber. I had already interrogated him several times during my visits without success as to his motives for such strange conduct, when one day, appearing not to observe him, I asked an attendant what caused the numerous dirty spots and holes in the wall. To our great surprise the patient broke the long silence which he had kept up to that day, in

* This article, a previously unpublished memoir, is incorporated in the last (3rd) edition of M. Brierre de Boismont's classical work on Hallucinations.

order to reply: 'Do you call those dirty spots and holes? Do you not see that they are Japan oranges? What delicious fruit, what colours, what odour, what admirable flavour!' Having said this he set himself to suck and lick with redoubled ardour. Thenceforward all was explained, and the poor man, whom till then I had pitied as the most miserable being, was, on the contrary, very happy, since most agreeable hallucinations of the senses of sight, smell, and taste procured him continual enjoyment."

The frequent occurrence of hallucinations, in association with madness, has induced several physicians to seek to estimate the proportion with greater precision than had previously been arrived at. Out of 145 patients under treatment at Bicêtre, M. Baudry says, 56 exhibited hallucination.* MM. Aubanel and Thore, in their statistics of the same hospital, say that out of 181 maniacs they found 70 cases of hallucination and illusion; that out of 66 cases of monomania 35 presented hallucination, and that amongst 21 individuals affected with lypemania 11 evinced false sensorial perceptions.†

A German author, M. Blumröder, whom I know only by an attack upon my work, *Des Hallucinations*,‡ has reproached me with not having made any addition in seven years to my 62 cases of hallucination of the first edition, and for having said that in the special form of mania hallucinations are *almost always* present, whilst, indeed, they are *never absent*. Convinced that the criticism of an adversary, be he even anti-catholic, is always useful, I have turned this to my advantage. Adding the 285 observations of my predecessor, M. J. Pressat, the younger, a conscientious physician, to 861 which I have collected in the space of twelve years (1848 to 1859), I have attained the respectable figure of 1146 facts, out of which I have noted 725 cases of hallucination and illusion. In spite of my efforts to conciliate the goodwill of M. Blumröder, I confess that the 229 cases of mania have only given me 178 examples of hallucination, which leaves a deficit of 51 cases! I proceed to detail the result of my researches. The following is a classification of the cases I have collected:—

| | Observations. | Hallucinations and Illusions. |
|---|---|---|
| 1 Acute delirium . . . . . . | 32 | 25 |
| 2 Mania . . . . . . . . | 229 | 178 |
| 3 Lypemania (melancholic monomania) . | 303 | 248 |
| 4 Hypochondriacal monomania . . | 63 | 33 |
| Carried forward . | 627 | 484 |

* *Thèse*, p. 14. 1833.

† *Recherches Statistiques*, p. 98 et suiv. Paris, 1841.

‡ Blumröder: *Jahrbücher der in-und ausländischen medicin*. No. iii. Leipzig, 1853.

| | | Observations. | Hallucinations and Illusions. |
|---|---|---|---|
| | Brought forward | 627 | 484 |
| 5 | Simple Melancholia | 1 | 0 |
| 6 | Stupor | 6 | 2 |
| 7 | Melancholic monomania with stupor | 7 | 3 |
| 8 | Monomania | 34 | 29 |
| 9 | Dementia | 82 | 41 |
| 10 | General paralysis | 147 | 37 |
| 11 | Feebleness of intellect, imbecility, idiocy | 53 | 17 |
| 12 | Alcoholic insanity | 73 | 49 |
| 13 | Hysterical insanity | 14 | 9 |
| 14 | Epileptical insanity | 28 | 9 |
| 15 | Puerperal insanity | 23 | 18 |
| 16 | Insanity of double form (*Folie à double forme*) | 27 | 14 |
| 17 | Moral insanity | 5 | 1 |
| 18 | Insanity | 19 | 12 |
| | Totals | 1146 | 725 |

*Summary.*

| | |
|---|---|
| Observations | 1146 |
| Hallucinations and Illusions | 725 |

The *extensive* examination to which we have subjected all these hallucinations convinces us that we should fall into fastidious repetitions if we were to give a summary of the 18 forms under which they were presented to our observation; we shall therefore limit our examination to four groups: acute delirium, mania, lypemania, and monomania; and we shall only offer a few remarks on such of the other groups as possess some special physiognomy:—

I. *Of the Hallucinations and Illusions in Acute Delirium.*

Acute delirium, which some physicians, and amongst others M. Pidoux, consider as acute insanity, on account of the violence of its symptoms and the continuity of the delirium, permits with difficulty the separation of hallucination from illusions. Abandoned without control to extreme impressions and internal sensations, attention being entirely wanting, the brain can but submit to and be led away by them.

Our observations number 32, and of these 25 were complicated with hallucinations and illusions, subdivided as follows:—

*Hallucinations.*

| | |
|---|---|
| Of Hearing and Sight | 5 |
| Of Sight | 1 |
| | 6 |

*Hallucinations and Illusions.*

| | |
|---|---|
| Of Hearing and Sight | 10 |
| Of Hearing, Sight, Touch, Smell, and Taste | 2 |
| Of Hearing, Sight, and Taste | 1 |
| Of Hearing or Sight | 1 |
| | 14 |

*Illusions.*

| | |
|---|---|
| Of Sight and Hearing | 3 |
| Of Sight | 1 |
| Of Touch | 1 |
| | 5 |

*Total.*

| | |
|---|---|
| Hallucinations | 6 |
| Hallucinations and Illusions | 14 |
| Illusions | 5 |
| | 25 |

This species of febrile maniacal insanity, by the intensity of the general excitement which it determines in the nervous and sanguine elements, ruptures the harmony of the intellectual and physical world; ideas take form, objects become metamorphosed, and the mind lives in the midst of a continual phantasmagoria, the scenes of which are, for the most part, sad. It is to this disposition of the mind, indicated in our work on acute delirium, that we should attribute the suicides so frequent in diseases called cerebral or inflammatory fevers, and which are generally none other than acute delirium. No doubt, also, the terror, the cries, the desire to bite and strike, are determined by hallucinations of a terrifying nature.

Simple hallucinations were observed in six individuals, and hallucinations associated with illusions in fourteen. In five illusions existed alone. The forms of false perception most frequently manifested were those of hearing and sight united; then come the perversions of smell, taste, and touch; and lastly, those of all the senses.

Hallucinations and illusions are in this malady the accessories of a delirium almost always general, in the midst of which a predominant delirious conception frequently shows itself. The mobility, confusion, and interlacing of ideas often renders the observation of these sensorial perceptions a matter of difficulty. It is without contradiction one of the forms of insanity in which they are most fugitive, and the impressions which they produce on the mind are often painful. Figures take the aspect of enemies and monsters; voices give the sound of menacing words; beverages have a detestable taste, and convey the impression that they are poisoned. Five of these unfortunates, under the influence of their painful sensations, made attempts at suicide; and two others attacked their attendants.

Patients suffering under acute delirium should be the object of continual attention, on account of the numerous illusions by which they are subject to be attacked. One fact which came recently under my notice shows that keepers are not always sufficient to prevent accident, and that, under some circumstances, it is necessary to have recourse to coercive measures. A young lady was brought to my establishment in violent delirium, with a continual tendency to suicide. Two servants were stationed in her room; and, on one occasion, a superintendent happened by chance to be there with them, when in a moment the patient seized the cord of her bed-curtains, passed it tightly round her neck, and, burying her body between the pillows, all their efforts to make her loosen her hands were unavailing. The peril was imminent; the superintendent, however, by a happy inspiration, struck her on the neck; the patient thereupon lifted her head, and this movement was just sufficient to permit a hand being slipped in, and so to stop strangulation. The mark due to the constriction presented, for several days, a red surface, two centimetres wide, in the anterior part of the cervical region. Whenever the patient was free she seized a favourable moment to renew her attempt.

The nature of the hallucinations in this form of delirium has often relation to the habits, character, and passions of the individual. A very religious young lady continually cried, "I am surrounded by demons; begone, Satan! my kind Jesus, drive him away!" A Protestant minister, who had suffered a great reverse of fortune, was subject to intermittent acute delirium. In all his accesses he saw sinister figures threatening him with hell, making wicked proposals to him, and otherwise tormenting him.

Illusions, either alone or associated with hallucinations, are very common in acute delirium. Figures change or take extraordinary aspects. Beverages are rejected with horror, because the patients believe that they are smoked, that their taste is detestable, and that it is sought to poison them. Some, on the contrary, fancy that their barley water has the flavour of the finest wines, and go into ecstasy while drinking it.

### II. *Of the Hallucinations and Illusions of Maniacs.*

The agitation of the maniac, his want of attention, and the mobility of his ideas, are so many circumstances opposed to a consecutive observation of his sensorial impressions. Nevertheless, the longer interval between the intermissions, the kind of connexion which glances out of the incoherence of his talk, the peculiarities of his actions, and the frequent presence of the patient, permit of a more attentive study of the disorder of his senses than in the case of acute delirium. One is led *à priori* to the conclusion that the general excitement should give rise to numerous false appreciations respecting external sensations; this agrees with observation. The establishment of hallucination requires a certain fixity of ideas, which only exists in monomania, and more particularly in melancholy monomania. Georget has remarked that hallucinations are rare in mania. The following extracts will show that they are more common than that distinguished alienist believed, but that simple illusions, or illusions combined with hallucinations, are the predominant sensorial symptoms.

The observations which we have collected of cases of mania number 229; and of these 178 presented complications of hallucination and illusion; the remaining 51 cases did not exhibit false sensorial perception.

The 178 cases are subdivided as follows:—

*Hallucinations alone.*

| | |
|---|---|
| Of Hearing and Sight | 26 |
| Of Hearing | 15 |
| Of Sight | 9 |
| Of Hearing and Touch | 2 |
| Of Hearing, Sight, and Touch | 1 |
| Of Hearing and General Sensibility | 1 |
| | 54 |

*Hallucinations and Illusions.*

| | |
|---|---|
| Of Hearing and Sight | 24 |
| Of Hearing, Sight, Touch, Smell, and Taste | 12 |
| Of Hearing, Sight, Touch, and Smell | 8 |
| Of Sight | 5 |
| Of Sight, Hearing, Smell, and Taste | 5 |
| Of Hearing, Sight, and Smell | 5 |
| Of Sight and Smell | 3 |
| Of Hearing, Sight, and Touch | 1 |
| Of Hearing, Taste, and Smell | 1 |
| | 64 |

*Illusions alone.*

| | |
|---|---|
| Of Sight | 27 |
| Of Hearing and Sight | 21 |
| Of all the Senses | 10 |
| Of Touch | 1 |
| Of Smell and Taste | 1 |
| | 60 |

*Total.*

| | |
|---|---|
| Hallucinations . . . . | 54 |
| Hallucinations and Illusions . | 64 |
| Illusions . . . . . | 60 |
| | 178 |

In the remarks we are about to make on the false sensations, we shall follow the classification of the five senses; the disorders of general sensibility shall be examined when we treat of melancholic monomania.

The hallucinations of hearing in maniacs consist of words most frequently of a jeering, derisive, reproachful, disagreeable, or threatening nature; in other cases they may be of a totally different character, leading to extreme contentment: the voices follow the maniac everywhere, leaving him no repose; the result is excitation, passion, agitation, scenes of violence or disordered joy. In the silence of night the crises are generally due to hallucination, whilst during the day they are more often determined by illusions. Many patients have conversations with invisible beings. In some cases the voices emanate from God or heaven, but the discourse they hold has no relation to their lofty origin; sometimes they announce to the maniac that he is an important personage—a prophet, a king, the master of the world, &c. The nature of the delirium deprives the voices of all consistency, so that they utter the most startling contradictions. These are two distinctive features of maniacal hallucination.

These voices may come from all directions, from above or below, from the courtyard or the street, from the belly or the stomach; they are loud and piercing, as spoken through a trumpet, obliging the patient to stop his ears, or sometimes they are confused, scarcely perceptible—like a whisper, silent and internal. The patients turn rapidly towards the place whence they proceed and pay great attention; they reply to them, speaking aloud, singing, or murmuring to themselves. Many hear voices of different intonation. The conversations are more frequent and noisy during the night. Other patients instead of replying by words make telegraphic signals to the voice in the air.

It is not unfrequent, when the voices are threatening, to hear maniacs call the guard to take the assassin; but these manifestations are more often produced under the influence of disordered sight.

Although external tumult contributes to diminish the intensity of the voices, one frequently meets with inoffensive maniacs who hear them in the midst of a crowd. A physician assured us, that for some years he had constantly heard voices calling him names in the streets and public walks of Paris, and addressing him with disgusting remarks. He walked in vain for hours together, they pursued him wherever he went. For some months particularly they surrounded him, and abused him without a moment's respite. "There," he added, "even at this moment they call me *pochard.*" On one occasion a military gentleman, convalescent of a maniacal affection, stopped in the midst of a conversation to say, "They call me a traitor; I am not."

Hallucinations of sight, like those of hearing, although more clear than in acute delirium, being very often mixed with and masked by illusions, are not always easily distinguished. They may be produced by all kinds of ideas, and the more fugitive the impression the shorter they last; if the emotion be deep the hallucination may persist some time. A farmer's wife subject to a nervous affection was attended for a month by a woman, whose sole treatment consisted in praying for her. This system of medication gave rise in the patient to an hallucination, showing her guardian angel continually in prayer at her side. One lady saw in a dream her friend, whose profession had obliged him to return to Africa. He related that he had fallen in battle and offered her his hand; she touched it and found it cold. The shock was so great that she started from her sleep and was attacked with mania. The impressions are often disagreeable, painful, hideous, and terrifying. One of our patients saw a serpent always ready to dart upon her and tear her in pieces.

The apparitions of mania, though sometimes distinct, are more commonly confused, imperfectly conceived, and quickly replaced by others: the rapidity of their succession may be such that the patient may believe he is looking at a phantasmagoria. One said to us, "I live in a world of dreams."

Illusions are much more frequent than hallucinations because the attention no longer presiding over the operations of the intellect, the brain is overrun by all sorts of impressions, external and internal.

The illusions of hearing have very various origins; words, noises, songs, are interpreted by the maniac in the most eccentric manner, who imagines that all these auditory sensations relate to himself. These impressions are the cause of unusual, sudden, and unforeseen actions. A young lady, who continually replies to voices, believes that the insults and abuse which she hears are offered by persons present with her, and when one is paying her the least attention, she slaps the face of the last speaker; or sometimes she will inflict this chastisement on others who have not opened their mouths, and if reproached for this behaviour she justifies herself, saying, "I was insulted without provocation, and therefore I take my revenge." Here, the hallucination and illusion supplement one another in the same patient. Another lady, equally tormented by hallucinations and illusions of hearing, whenever one or other of these impressions takes hold of her, gets into a fury with her fancied insulters, and loads them with epithets so ill-sounding and so little in harmony with her education, that one is constrained to conduct her immediately to her room. Many of these patients cannot remain in one place because of the noise which is made wherever they find themselves.

The illusions of sight play a considerable part in mania. One of the most common is the change of persons. Out of 124 cases we have observed this in 62. Sometimes the patient sees in the assistants his relations, friends, and acquaintances; sometimes he believes himself surrounded by hideous figures, evil-disposed persons, and enemies. Very often these figures make faces at him and mock him; he fancies that there is a conspiracy against him, and that all the patients of the establishment are persons suborned to take their part in it. One of our patients fancied that his messmates were so many people that he had known; he held fragmentary conversation with them in accordance with their supposed positions. These transformations of figure give rise at times to very moving scenes. A lady was in the habit of asking daily, in most pathetic tones, to see her husband and her son; she would take no nourishment, and it became necessary to feed her by means of the œsophagus tube. I had information that the same requests had been made in another establishment, and that the reunion so ardently desired had produced no effect. Nevertheless, touched as I was, like others in the house, by grief apparently so genuine, I caused the husband and the son to come and see her; in spite of my experience I still hoped. After looking at them, the unhappy woman groaned deeply and cried, "These are not they!" The experiment was made a second time without better success; it was not again repeated, as it might have been attended with painful consequences to the child.

These changes are not limited to substitutions of persons; on several occasions patients have told us that the speakers were dogs, geese, horses, or monkeys. Amongst the dangers to which one is exposed with lunatics, those resulting from these illusions of hearing and sight are not the least. The persistence of fancied insult, and the constant presence of an enemy, have been the immediate cause of more than one tragical event.

The mobility and rapidity of visual sensations produce the most varied combinations. Everything about a maniac changes each instant; around him accumulate a crowd of objects, each of which has to him a special significance; every gesture which he sees is a step in some intention which concerns him. A patient assured us that passers-by, coachmen, horses, and carriages all assumed singular postures and strange attitudes towards him.

Inanimate objects undergo the same metamorphoses: pebbles, leaves, rags, are precious stones, rare substances, or magnificent paintings; lines and bars are portraits and pictures, &c.; dust, salt, and pepper become poisons sprinkled over the food, which is then obstinately refused. A maniac looks at a star, and he discovers God there; soon he sees him in six other stars, and fancies that he is the object of his attention. This influence of the stars is much more commonly believed by reasonable people than is generally imagined. We have known several very intelligent, but very nervous persons, who, when sick or moved by some important event, would rise during the night, look at the sky, and mentally address some particular star, as though it were their mediator with God.

In illusions of the sight figures and objects enlarge and diminish, remove and approach, colour, break, and become pale, as in hallucinations. The auditory sensations of illusion also present the same phenomena which are observed in hallucination of hearing. The voices are sonorous, confused, whispered, various, far off, near at hand, or internal, &c.

Finally, the illusions of sight may relate to the patients themselves, and give rise to errrors of personality. Thus, there are maniacs who believe themselves metamorphosed into animals; there are others who imagine themselves growing visibly, who fancy they see their hair lengthen, their head inflate like a balloon, &c.; others believe themselves to become very light, so as to rise in the air and fly, &c.

The illusions of touch and of smell are much less frequent than the preceding, although still sufficiently numerous. It is difficult to distinguish them from hallucinations on account of the mobility of maniacal ideas and impressions; this difficulty is even manifest in the case of melancholic monomaniacs whose intellect is only deranged on a small number of subjects.

Maniacs subject to illusion of touch say that they are beaten. They are convinced that they are pinched or burned. Several believe themselves magnetized and feel commotions. The women maintain that they are the victims of all kinds of attempts; that people touch them, violate and otherwise abuse them.

One lady assured her husband that Satan slept with her every night. On the other hand, there are some who take pleasure in these voluptuous sensations. It is to be remarked that lunatics who abandon themselves to this indecency of the imagination are often women of exemplary conduct and very reticent in their language.

The illusions of smell manifest themselves most often by the sensation of bad odours; at least the cases of this nature are most common; some patients, however, insist that they inspire perfumes. The impressions of taste are also perverted; maniacs persuade themselves that their food is detestable, and above all that it is poisoned; the refusal of drink and nourishment is not unfrequently observed when these two illusions are united.

The difficulty of distinguishing between illusions and hallucinations is often increased by the fact that, in the former cases, real sensations frequently exist which are transformed by the imagination of the patient. Thus rheumatic and nervous pains cause the patient to fancy that he is beaten and illtreated; a bad breath or foul taste suggest to him the idea of infected odours and poisoned food.

Hallucinations and illusions of mania may appear in the first stages of the disease or precede it; they may complicate it and terminate with it, or they may remain after it has passed away.

It sometimes happens that illusion passes into hallucination, and *vice versâ;* we have already quoted examples of this. A maniac will fancy that he sees a grim beast in the persons of those who come before him, and afterwards, by a process natural to man, he detaches the image from the idea, places it before his eyes, and then, terrified at his own creation, shrieks out and gives himself up to furious combat with the imaginary animal. At other times these maniacs, after having taken strangers for a person with whom they are acquainted, see this person before them, speak to him and receive replies. These changes are also observed in other forms of insanity.

Hallucinations like mental diseases may be symptomatic. A woman suffered from a serious disease of the intestines: her reason wandered, she shrieked and sang and talked most incoherently. In the midst of her delirium she imagined she saw in the court great fishes, for which she angled with a line. At times she was greatly afraid because the fishes were going to eat her. As the intestinal affection got better these foolish ideas diminished in intensity, and when she left us she was entirely cured. We have observed similar facts in diseases of the heart, and Dr. Forbes Winslow has reported several examples in his work *On Obscure Diseases of the Brain.* These symptomatic hallucinations and illusions may be caused by many other diseases.

The hallucinations and illusions give rise to the most singular disorders.

Mademoiselle O—— was remarkable for her judgment, the rectitude of which was such that she was constantly consulted by her friends. This fact, which was testified to me by a great number of persons capable of appreciating her character, proved to me that if the absence of judgment be one of the distinctive features of insanity the rule is not without exception. Besides, who does not recall the case of a man whose powerful reason aided in the preservation of the peace of the world, and who, nevertheless, in the prime of life became the subject of mental alienation?

The first symptoms of evil manifested themselves as by a kind of revelation; she prayed her parents that in case she should ever become insane, they would send her to an establishment which she named. This request greatly surprised them, for at that time she conversed reasonably and had evinced no peculiarities.

Mademoiselle O—— fancied, in the first instance, that she heard voices abusing her. They threatened to quarter her, to make mincemeat of her, to eat her, &c. They commanded her to swallow all that came before her. Obedient to their order she introduced successively into her stomach, earrings, pins, and mittens, and she would have done the same with a box of dominoes, but that, suspicion having been roused, her request for them was refused. She laughed or went into a fury when any one made an observation to her, and sought to strike or scratch, fancying they were devils.

Her conversation, which was full of incoherencies, indicated the disorder of her faculties; some one was coming to take her away to China; the devils loaded her with illtreatment and spoke obscenely to her; we were Messieurs so-and-so, and then our figures changed into those of bandits and ruffians. By a sudden and incomprehensible transition these foolish ideas disappeared, as though the wind had swept them away, and a sensible and instructive conversation struck the persons present with astonishment, such a rapid change being inexplicable.

This young lady also experienced a perversion of cutaneous sensibility, which caused her to find pleasure in tearing away her skin, a symptom met with in a great number of lunatics, especially amongst melancholics and idiots. This mania was sometimes carried so far that we have counted as many as twelve large ulcerations on different parts of her body.

Toil and fatigue, and the critical period, appear to have been the cause of the mental affliction of this young lady.

There were days when she was convinced that she wasted and shrunk perceptibly to the eye, although her corpulence was enormous. Once she begged a lady to put her in her umbrella or her bonnet in order to carry her away the more easily. At other times she believed she was metamorphosed into a cat or a dog, and would counterfeit the cries of those animals for hours together.

These illusions were replaced by another, which lasted a long time. She complained that every one accused her of being a man whilst she was a woman, and to give convincing proof of this fact she would expose her breasts and lift up her dress. But these acts did not stop there; this young lady, so chaste and modest, in whom religious principle, well-understood and severe morality, should have conquered or at least stemmed back the animal instinct, abandoned herself to onanism in the most ungovernable manner. We have often seen her give way to this manœuvre in the presence of several persons without manifesting the slightest shame, and what is still more remarkable, though analogous to what is met with amongst reasonable persons, she would afterwards commence to converse calmly, as if nothing had happened.

It has been said that the genetic instinct has its seat in the cerebellum; but how comes it that this organ wakes up at the period when its functions are about to cease? According to a physiological law, should not a long inaction have induced atrophy? We once had under our care a lady perfectly well brought up, very religious, the mother of several children, who had never read romances, but who nevertheless when she arrived at her critical period was assailed with most imperious desire, and recoiled from no method of excitation so that she might satisfy it. Order was completely restored when the symptoms were allayed. How can we attribute to the cerebellum the uterine furor which developes itself in mothers of families and virtuous women immediately after the appearance of an eczematous eruption on the generative parts? Enough, however, on this subject, which conducts us naturalty to another reflection of the same order. One is often asked how it is that the filthiest language is used and most lascivious actions committed by women well brought up, whilst those who have been foolish with their bodies maintain on the contrary a reserved demeanour? The reply, it appears to us, should be found in the organization itself. It is possible, by education and religion to quell and stifle an instinct, but to destroy it, never. More than that, it returns the more impetuously if not satisfied. This is the reason why in religious lunatics the sexual passion shows itself in all its force.

Mademoiselle O—— remained a full year in this state of mania, a prey to hallucinations and illusions, and perpetual agitations. Sometimes she saw devils and heard voices; at others she saw figures of friends who came to visit her, or she was served with poisoned food, or her room was filled with infected smells, or some one made a great noise to prevent her sleeping. Sometimes she pretended we had beaten her, and exhibited the ulcerations she had caused by tearing her skin. At times she fancied that she was the Duchess de Berri, that her husband had exercised his rights, and that she was confined of a son. When this idea entered her head she sought all over for her child, and fancied she saw it in every object. After this lapse of time we observed that she became more calm, and that her conversation presented long lucid intervals. She was permitted to walk in the garden, and she no longer stripped off her clothes. The improvement continued, and she ultimately recovered her senses completely. It has been contended, most erroneously, that after crises of such length there always remains some alteration in the intellectual faculties. If this were true, Mademoiselle O—— must have been an exception to the rule, for she passed her entire days with us, which she divided between conversation and lessons which she gave to my children. The clearness of her explanations, the choice of her examples, and the excellence of her method, daily excited our surprise. Her memory was prodigious, and nothing had been forgotten in her long night of gloom. During ten whole days this miraculous resurrection was maintained; but gradually foolish and strange ideas again passed through her brain. She stopped in the middle of a most sensible conversation to say that she was not a Chinese, that she had never been in Africa, and had never cut anybody's throat. Sometimes she suddenly raised her dress to prove that she was not a man. The disorder reappeared in all its intensity; and from the time of this relapse up to her departure, which took place four months afterwards, she experienced intermittences of calm and unreasonableness. In her accesses she undressed herself to show that she had changed her back, that she was an animal, or Mademoiselle B——, one of the patients of the house, or to prove the contrary. Another peculiarity of this case was, that Mademoiselle O—— often wrote in the midst of the most incoherent discourse letters that did not contain a word that could reveal the state of her mind, and which on an inquest would have been presented as so many proofs of the excellence of her judgment.

The hallucinations and illusions of maniacs lead to conclusions and actions which at first seem often incomprehensible, but which a well-grounded acquaintance with these two orders of symptoms will almost always serve to explain in a natural way. A lunatic looks at you with a furious air, he rushes to strike you: an illusion has changed your figure to that of an enemy, or he fancies that you are making grimaces at him or insulting him. Another will jump out of the window because the street appears to him on a level with the room, or that he thought to pass into a garden filled with fruits and flowers. Another throws his bread into a brook or stamps it under his foot, in order to render it more tender and give it a flavour which it does not possess.

Many maniacs refuse food on entering an asylum, believing it to be poisoned. Some look up to the sky with ecstasy, because the clouds are of gold, or appear to represent horsemen or palaces. One of these patients was always turning round on his heel: he had been an engineer, and it was ascertained that for many years, while in the establishment of Dr. Blanche, he had an idea that he could raise water to an immeasurable height by means of this rotatory movement.

Some see insects, animals, or startling colours on their apparel. The slightest noises occasion the most various impressions; they fancy they hear threats or concerts, or that cannon are being fired.

These false sensations are often the cause of hurtful or dangerous actions. Some maniacs kill because they see the devil before them; others die of hunger, commit acts of incendiarism, or mutilate themselves, because they obey a commandment. Instances of this kind are very numerous. These eccentricities, though often difficult to trace to their true causes, by reason of the agitation or irascibility of the patient, or the impossibility of getting him to answer, are nevertheless to be accounted as due to hallucinations and illusions.

*Summary.*—The hallucinations of mania are more frequent than those of acute delirium, but illusions, either alone or combined with hallucinations, are the predominant sensorial phenomena.

— The preponderance of illusions is due to the circumstance that, although the cohesion of idea is more decided in mania, the faculty of attention is seriously impaired, and the imagination is therefore continually the sport of external impressions.

— The hallucinations and illusions of mania are often numerous, continual, confused, and changeable.

— The most frequent are those of hearing and sight. Hallucinations and illusions of all the senses are more frequent in this form of madness than in others.

— It is not always easy in cases of mania to distinguish hallucinations from illusions, not merely on account of the mobility of the impressions and want of attention, but also because of the existence of real sensations.

— The want of fixity in the impressions produces the most perplexing oppositions and contradictions; the hallucinations and illusions themselves possess also a character of general incoherence.

— The physiology of hallucinations and illusions furnishes materials for their clearer comprehension.

— The frequency of illusions in mania is due to the want of attention.

— Any kind of noise may give rise to illusions of hearing; those of sight are more specially characterized by changes of person.

— The illusions of touch, smell, and taste are generally of a painful and disagreeable nature.

— Hallucination and illusion may precede and give rise to mania, or may be replaced by it, the latter being but a transformation. Illusions change sometimes to hallucinations and *vice versâ*.

— Hallucinations, though generally primitive in mania, are sometimes symptomatic.

— Hallucinations are of great importance from a medico-legal point of view, for they are often the cause of acts of violence, attempted theft, incendiarism, murder, suicide, &c.

(*To be continued.*)

---

